# The challenges and necessity of situating ‘illness narratives’ in recovery and mental health treatment

**DOI:** 10.1192/bjb.2021.4

**Published:** 2022-04

**Authors:** Nagina Khan, Derek K. Tracy

**Affiliations:** 1 Touro University Nevada, USA; 2 Oxleas NHS Foundation Trust, UK; 3 King's College London, UK

**Keywords:** Mental health, personalisation, recovery, self-management, illness narrative

## Abstract

In mental health services, recovery constitutes a guiding principle that is endorsed in professional medical guidelines and has become central to mental health policies across the world. However, for many clinicians, it can be a challenge to effectively embed recovery concepts into professionally directed treatment of disease without distortion, and ostensibly away from what matters to those who use the services. We discuss the evolving and multifaceted concept of ‘recovery’, including illness narratives to frame our discussion. We demonstrate how integration between a person-directed management of illness and a professionally directed treatment of disease can converge, resulting in positive outcomes for people with mental illness.

Increasingly, patient-led research has been challenging traditional professional perceptions on the trajectory and outcomes of mental illness. (Of note, ‘patient' is the stylistic word choice for this journal; the authors had submitted ‘service user' in the draft. We recognise this is an area of discussion and debate). Many people may not fully recover from mental health problems, but can discover how to cope with their condition. Evidence has also highlighted that recovery, and many of the processes involved in it, can happen across a period – in many cases, outside of formal medical environments. In this editorial, we summarise and discuss contemporary aspects of recovery, including the co-concept of ‘illness narratives’. We argue that although recovery can be a contested topic with differing views, it is also ubiquitous, and its meanings are not always fully incorporated into truly co-produced models. Models that put patients’ narratives at the core could perform as a connecting mechanism that provides a process to take account of ‘personalisation’, which is likely to create a better fit with individual context, structure and the complex diverse realities of recovery-oriented practice and routinely provided interventions.

## Definitions and debate

People outside of the formal health settings understand recovery in terms of their individual, specific and personal perceptions. Accordingly Anthony has shown that illness can hinder a person's perception of a productive life and undermine their process of recovery.^1^ Anthony's work remains highly influential (and cited) to definitions of recovery, stressing that ‘recovery is what people with disabilities do, recovery is a truly unifying human experience’.^1^ He also asserts that recovery, is ‘multi-dimensional, defying simplistic measurement’.^1^ Other perspectives on recovery have been explored: Slade has divided these into two forms, with clinical recovery being defined as symptom changes viewed by the clinician,^2^ and ‘personal recovery’ being defined as what the individual narrates and understands in a meaningful way.^2^

What is strikingly obvious here is that recovery is being presented in a distinct and separated pattern. This separation appears to be divided by the focus and the lens through which it is viewed. It is questionable whether this separation is helpful both clinically and furthering the discourse on recovery itself. The separation pushes apart the idea that ‘professionally directed’ treatment and ‘person-directed’ treatment can converge; indeed, the spirit of recovery would challenge the language of this – ‘directed’ inferring ‘being done to a person’ and would call for it to be more fitfully replaced by ‘perspective’. McWade also reinforces our view that this concept of recovery can be defined in such a way that allows freedom to converge medical expertise and affords patients the autonomy in their individual journey to recovery.^3^ Patient movements have dominated the concept of recovery since the mid-1970s,^4^ and currently there has been criticism of professionals that look to be trying to ‘mainstream’ recovery concepts to their advantage.^5,6^

### Research on recovery

There has been a significant amount of research and published work attempting to support each of the individual recovery concepts, but little has been done on the positive outcomes of converging the constructs. Recovery research ranges from personal accounts^7^ to attempts to define, standardise or measure the conceptual framework,^8,9^ alongside histories^10^ and empirical studies.^11,12^ Recovery-orientated practice guidelines exist for service provision,^13–18^ including an emerging critique^19^ resting on personal recovery,^9,20–22^ recovery-oriented services,^6,23–29^ in addition to provider competencies.^25,30–36^ Despite the accumulation of research produced to support or isolate contested elements of recovery, disagreement and criticism remains over the definitions of recovery and on whether recovery has to be survivor-led or a policy directive.^37^

Narrative and systematic forms of syntheses and reviews have tested tools^20,22^ to incorporate accepted components of recovery.^34,38^ This, however, has only led to further compound the idea of mainstreaming of recovery.^37^ Amid this accumulation of criticisms, Braslow has shown that ‘recovery is everywhere’ despite including an array of perspectives.^5^ Since the 1990s, the concept of recovery in mental health domain has affected both the person experiencing mental health difficulties and the clinicians.^37^ A conceptual framework that acknowledges recovery^33,36^ has been produced by Leamy et al, highlighting five main approaches in their CHIME framework: Connectedness, Hope – optimism, Identity, Meaning – purpose, and Empowerment.^38^ However, CHIME has not been without its critics. Bird et al carried out a validation study applying thematic analysis of data from CHIME, using focus groups with those with diagnosed schizophrenia, bipolar disorder and depression.^36^ They demonstrated that the CHIME framework was both valid and appropriate for use, but highlighted areas that were not included, such as ‘a desire for practical support, issues around diagnosis and medication, and scepticism about the concept of recovery’.^39^ As noted, evaluating recovery is an inherently complex concept,^40^ challenging the essence of creating ‘reliable measures of individual recovery’^39^ and the construct validity of this. Andresen et al suggest that if the said concept of recovery is entirely individual,^41^ contrary to ‘what can it be normed and as might be expected for a concept in which symptom reduction is not paramount’, then ‘correlations with customary clinical outcome measures may possibly be poor and convergent validity low’.^42^

### Embracing diversity

Given all of this diversity of recovery principles, it is perhaps not surprising to find professionals misunderstanding and confused^40,43^ in the operational aspects, emphasised in popular guidelines, discourse, policies and research. Pilgrim aptly suggested that recovery is a polyvalent concept.^43^ However, this does not translate easily to those who may wish to activate the recovery construct for better patient outcomes and improved service delivery. Quiet so, Braslow has argued that recovery remains an ‘unquestioned overarching principle and popularly known to include a melange of beliefs, values that materialized because of the intellectual and social movements’.^5^

## Moving to recovery-orientated services

### An anthropological model

To further link back to the discussion on the concept of converging the person-directed management of illness and professionally directed treatment, we recognise, similar to Davidson and Roe, that an anthropological model – one situated upon ‘principles of reciprocity’ that concerns itself more with the integration of healthcare^40^ – could complement and augment professional knowledge.^44^ However, this requires a substantial shift in the clinical lens, incorporating ‘lay types of knowledge’, such as a person's own understandings of dealing with illness, including social functioning.^44^ Suitably, Davidson and Roe conceptualise that meaningful illness narratives are a gift exchange, providing meaning, emotive steadiness, narration and collective experience.^40^

### Illness narratives and professionals’ treatment

Biological aspects have often been viewed by patients as both coercive and impersonal,^45^ although we may be seeing some welcomed change with this, and COVID-19 may offer an interesting corollary, with the emphasis of ‘long-COVID’ care moving toward wellness and self-management of long-term chronic difficulties. Pilgrim has pointed out that the root basis of patients’ adverse perceptions can be based on pathology, making clinicians appear as ‘chemotherapists with a prescription pad’.^45^

### The UK and co-production: the Care Programme Approach as an exemplar

In the UK, the National Institute for Health and Care Excellence explicitly calls for care plans to be jointly drawn up between individuals and their clinician, with shared decision-making and agreed dates to review its progress.^46^ The Care Programme Approach (CPA) necessitates that health and social needs are comprehensively assessed and reviewed with individuals with serious mental illness, with a ‘philosophy of recovery and to foster personalised care’.^47–49^

Despite their imbedding in contemporary British mental healthcare,^50^ there remains a general lack of data exploring actual practice in the community, and even less that is focused on in-patient care.^51^ The Healthcare Commission in the UK assessed in-patient performance across 554 wards in 69 National Health Service (NHS) Trusts. About 40% were rated as ‘weak’ when it came to including patients and carers; astonishingly, half of care plans had no evidence of recording patients’ views, and about a third made no mention as to whether there was a carer involved. A further third had input from the patients’ community care coordinators only some or none of the time.^52^

Work by Simpson et al examined the views and experiences of stakeholders involved in community mental healthcare, investigating factors related to the provision of personalised, collaborative, recovery-focused care.^53^ They found substantial variations among sites for results on therapeutic relationships and participant groups in their study, related to the experiences of care planning and understandings of recovery and personalisation.^53^ Consequently, carers expressed varying levels of input, and despite risk assessments being part of central clinical concerns, they were rarely discussed with patients.^54^

Patients valued therapeutic relationships with care coordinators and others, and saw these as central to recovery. However, in another study by the same team, the staff, patient and carer interviews revealed gaps between shared aspirations and realities,^55^ and staff accounts of routine collaboration contrasted with patient accounts and care plan reviews. They also found that personalisation was not a common phrase, but care was often delivered in an individualised way.^55^ McWade has argued that this endorses that perspective of failure of co-opting or mainstreaming the ‘thing’ that is recovery.^3^

### Contact with clinicians and patient engagement

‘Illness management’ can be understood as an approach to support patients with a diagnosis to engage with clinicians, to reduce patient susceptibility to the disease.^56^ Conversely, ‘illness problems’ are the principal difficulties that symptoms and disability create in lives, and ‘illness behaviour’ then consists of initiating treatment (for example, changing diet and activities, resting, engaging in exercise, taking over-the-counter or prescribed medications and deciding when to seek care from professionals).^57^ There is an overdue lack of understanding placed on the notion that there is a pre-existing underlying relationship between the person and their illness. Albeit in separation of formal treatment environments, this occurs in the way individuals perceive their difficulties, the type of help they envisage, and the approach they are willing to engage with for formal treatments; it includes making contact with clinicians and entering into a contract with services for future treatment.

### The socialisation of the practitioner

For the purpose of this editorial, a critical discussion cannot be complete without including a focus on clinicians understandings. Hitherto, psychiatric models have perhaps viewed the concepts of recovery from mental illness in a similar style to how clinicians have viewed physical illnesses.^58^ We propose that Kleinman's illness narratives model has proved influential in this regard, remarking how the practitioner has also been socialised into a distinct collective experience of sickness. It is true that clinicians are trained to capture the essence of illness by using concepts that delineate disease. For instance the use of expressions such as relapse, recurrence, remission and recovery.^59^ In this way, the symptom profile is used to transform the patient's illness (in a form of recasting of illness in accordance with the theories of disorder) into a disease formation.^57^ The absence of an ongoing intervention other than that relationship can be perceived by care managers as a need for premature discharge from services, for fear of creating dependency. However, some who use services desire an ongoing relationship, which commonly matters more than interventions, and this aspect can be missing in some recovery narratives. The construct of so-called ‘palliative psychiatric care’ is informative in this regard.^60^ Further, moral experience is central to Kleinman's model of illness narratives, incorporating constructs of ideal virtues of the practitioner^61^ and so opening up ‘illness narratives’ to create patient ‘storylines’, which brings to life inanimate parts of practices, policies and discourses.^62,63^ Hajer suggests that storylines are ‘narratives on social reality’, which combine elements from many different domains and ‘provide actors with a set of symbolic references that suggest a common understanding’.^64^ Albeit critically, Kleinman also envisaged that partnership is vital and is susceptible to change over time, with caregiving perceived as a construct focused more on ‘doing good for others in their world’ and projecting that ‘as earnest and naïve as it sounds, it is what medicine is really about’.^65^

### Patient perspectives of ‘corporate’ recovery/criticism

Despite the common understandings, it should be noted that some patients remain suspicious of the recovery concept because it is potentially intolerant of those who do not change, and so it may remain, in their eyes, a source of oppression used against them.^66^ There is a recurring critique of recovery presented as the ‘next best thing’,^67^ and a mere form of symbolism, undermining ‘authentic alliances’^68^ donning recovery in a sense to reduce effective support.^69^ Service cuts have been associated with the manner in which services and health systems manage future demand for mental healthcare, allied to the economic cutbacks planned for financial savings.^70,71^ Consequently, recovery concepts used in this method risk being used, or at least perceived, as indicative of justification to reduce services or their ability to provide timely input.

## Moving forward: making it work

In the majority of English-speaking countries^40^ the importance clinical recovery^2^ and personal recovery are touted in guidelines for key clinicians.^72–76^ The significance of tackling personal recovery, in conjunction with more standard concepts of clinical recovery,^2^ is currently endorsed in guidance for all key professions.^72–76^ Whether it as a model or framework, a movement or a guiding ethos, recovery is now ‘the hegemonic guiding principle of public mental health policy’.^5^ The social sciences have already reworked treating disease as a process of medical micro-encounters,^77^ and to the idea that ‘disease problems’ can occur within an individual's circumstances of everyday life.^76^ It is evident that researchers, clinicians and services alike may require a more complex approach to personal narratives and construction of meaning if individual recovery is to be more clearly understood.^78^

Psychiatric treatment historically conceptualised primarily based on a disease model could have the potential to impede the long-term treatment and assessment of those with chronic difficulties and illness. Linked to this assertion, Voronka has indicated that narratives can be utilised and shaped as a ‘gap-mending’ strategy,^79^ not merely because they interfere with professional knowledge, but because they have the power to strengthen the capabilities of individuals to bring forth personal goals and generate a sense of identity,^17^ a fusion, a convergence to create a mechanism of acceptability, of both person-directed management of illness and professionally directed treatment of disease, or, in essence, shared understandings of recovery and shared decision-making toward that outcome. Roberts and Hollins have also encouraged that medicine ought to discern that ‘disease understandings’ are embedded in patients’ experiences and their daily lives, and are considerably tied to a person's social history.^71^ This necessitates clinicians to move from a tradition of ‘paternalistic attitudes’ of helping or being the expert, to create meaningful alliances.^80^ Likewise, Lawton et al described an approach to illness experiences that recognises a dynamic interplay between ‘survivor’ and the healthcare system, whereby one affects the other,^71,77^ suggesting that medical encounters merely comprise a relatively insignificant portion of most patients’ lives (although symbolically they may represent more). Accordingly, the medical support to patients’ ‘sense-making’ tends, in reality, to be exhausted by the conditions and encounters met in the extra-medical social world.^81^

Recognising and embracing recovery concepts enhances care and the therapeutic experience for professionals and patients. The possible ‘recursivity’ between services and perceptions is relevant to understanding the way in which those with mental health problems might engage with care services.^73,81^ Peer support is increasingly recognised and implemented within NHS services, with roles in the UK typically at band 4/5; indeed, the Health Education England publication ‘Stepping Forward to 2021: The Mental Health Workforce Plan in England’ advocates even greater expansion of this.^82^ Nevertheless, there has equally been a call and need to deliver care in ways that have just not been possible in traditional teams. A potentially very fruitful and positive development in this regard has been the growth of recovery colleges, driven by strong patient engagement and roles, often at odds with, or at blurred boundaries to mainstream NHS services. Accordingly, central to improving outcomes for individuals, services should involve providing services that are ‘person-centred, strengths-based and recovery-focused’.^74^ To enable the interpretation of the recovery approach into practice, there is a necessity to involving illness narratives as a mechanism to personalise treatment and care so that it can be operated and assessed within medical and research environments.^20,25^

## Data Availability

Data sharing not applicable – no new data generated.

## About the authors

**Nagina Khan,** PhD PGCert BHSc, Senior Research Associate, College of Osteopathic Medicine, Touro University Nevada, Henderson, USA; **Derek K. Tracy,** FRCPsych, Consultant Psychiatrist and Clinical Director, Oxleas NHS Foundation Trust, Queen Mary's Hospital, UK; and Senior Lecturer, King's College London, UK

## Supplementary material

For supplementary material accompanying this paper visit http://doi.org/10.1192/bjb.2021.4. click here to view supplementary material
